# Long-term changes in serum levels of lipoproteins in children and adolescents with attention-deficit/hyperactivity disorder (ADHD)

**DOI:** 10.1007/s00702-022-02583-5

**Published:** 2023-02-24

**Authors:** Franziska Huber, Jan Schulz, Robert Schlack, Heike Hölling, Ulrike Ravens-Sieberer, Thomas Meyer, Aribert Rothenberger, Biyao Wang, Andreas Becker

**Affiliations:** 1 Clinic for Child and Adolescent Psychiatry and Psychotherapy, University Medical Center Göttingen, Von-Siebold-Str. 5, 37075 Göttingen, Germany; 2 Department of Epidemiology and Health Monitoring, Robert Koch Institute, Unit Mental Health, Berlin, Germany; 3 Department of Child and Adolescent Psychiatry, Psychotherapy and Psychosomatics, University Medical Center Hamburg-Eppendorf, Hamburg, Germany; 4 Department of Psychosomatic Medicine and Psychotherapy, University Medical Center Göttingen, Göttingen, Germany; 5 Partner Site Göttingen, German Centre for Cardiovascular Research, Göttingen, Germany; 6 Department of Clinical, Educational and Health Psychology, University College London, London, UK

**Keywords:** ADHD, Lipid metabolism, KiGGS study, Lipoproteins, Children, Adolescents

## Abstract

Attention-deficit/hyperactivity disorder (ADHD) is a neurodevelopmental disorder affecting approximately 5% of children worldwide. The causal mechanisms of ADHD remain unclear as the aetiology of this disorder seems to be multifactorial. One research field addresses the impact on lipid metabolism and particularly serum lipid fractions on the development of ADHD symptoms. This post hoc analysis aimed to investigate long-term changes in serum levels of lipoproteins in children and adolescents with ADHD and controls. Data of German children and adolescents from the nationwide and representative “Kinder- und Jugendgesundheitssurvey (KiGGS)” study were analysed at baseline and at a ten-year follow-up. At the two time points, participants in the control group were compared with those in the ADHD group, both before and after propensity score matching. Differences in total cholesterol, low-density lipoproteins (LDL), high-density lipoproteins (HDL) and triglycerides were assessed between matched children with and without ADHD. In addition, subgroups with versus without methylphenidate use were compared at both time points. At baseline before matching, there were no significant differences for lipid parameters between participants in the ADHD group (*n* = 1,219) and the control group (*n* = 9,741): total cholesterol (Exp(*ß*) = 0.999, 95%-CI 0.911–1.094, *p* = .979), LDL (Exp(*ß*) = 0.967, 95%-CI 0.872–1.071, *p* = .525), HDL (Exp(*ß*) = 1.095, 95%-CI 0.899–1.331, *p* = .366) and triglycerides (Exp(*ß*) = 1.038, 95%-CI 0.948–1.133, *p *= .412). Propensity score matching confirmed the non-significant differences between the ADHD and non-ADHD group at baseline. At the 10-year follow-up, *n* = 571 participants fulfilled complete inclusion criteria, among them 268 subjects were classified as ADHD. The two groups did not significantly differ in lipid fractions, neither cross-sectionally nor with regard to long-term changes. There was also no significant difference between methylphenidate subgroups. In this sample of children and adolescents we could not reveal any significant associations between serum lipid fractions and the diagnosis of ADHD, neither cross-sectionally nor longitudinally; even when methylphenidate use was considered. Thus, further studies using larger sample sizes are required to investigate putative long-term changes in serum lipid fractions related to ADHD.

**Supplementary Information:**

The online version contains supplementary material available at 10.1007/s00702-022-02583-5.

## Introduction

Attention-deficit/hyperactivity disorder (ADHD) with a worldwide estimated prevalence of 5% (Polanczyk et al. 2007) is characterized by three core symptoms, namely inattention, impulsivity and hyperactivity lasting for at least 6 months (Banaschewski et al. 2017a) which commonly results in impairments in private and professional life (Geissler and Lesch 2011). The diagnosis of this neurodevelopmental disorder is challenging, since about 75% of the patients are affected by a comorbid disorder (Banaschewski et al. 2017a), such as conduct disorder, depression, anxiety, tic disorders and learning disabilities (Göbel et al. 2018). Moreover, recent studies have underscored an increased risk for development of somatic disorders (e. g. cardiovascular diseases, asthma, headache, functional vision problems and obesity). Still, a direct connection could not be established so far and data suggest an indirect connection based on genetic factors (Garcia-Argibay et al. 2022; Arrondo et al. 2022; Xu et al. 2022; Kase et al. 2021; Robert Koch-Institut (RKI) 2018; Pan et al. 2021; Bellato et al. 2022; Li et al. 2022; Li et al. 2020). For a long time, ADHD has been classified primarily as a disorder of children and adolescents but recently it has been shown that also many adults exhibit persistent ADHD symptoms, even if they often do not comply with the full diagnostic criteria (Faraone et al. 2015). Despite great efforts on the part of research, so far, no single risk factor has been identified as specific for the development of ADHD suggesting that its aetiology may be multifactorial, and include genetic, psychosocial and environmental risk factors (Thapar et al. 2013). Alterations in the dopaminergic-noradrenergic neurotransmitter systems have been described suggesting structural deviations and functional dysregulation of monoamine pathways in the brain (Faraone et al. 2015). In the context of nutritional influences, especially the role of ω-3/-6 polyunsaturated fatty acids (PUFA) in ADHD patients has been investigated since animal studies had indicated that deficiency of PUFAs led to reduced neuron size, abnormal neuronal structure (Ahmad et al. 2002) and altered dopaminergic and serotonergic neurotransmission (Chalon 2006). Some studies have suggested that supplementation of ω-3-PUFA may provide additional benefits regarding symptom reduction in subjects with ADHD. However, many controversies on this issue remain (Hawkey and Nigg 2014; Agostoni et al. 2017). The German S3 guideline for ADHD currently reports a positive but very small effect of omega-3/-6 supplementation and does not recommend it for the supportive treatment of ADHD (Banaschewski et al. 2017b). Although in early literature, Maes et al. (1997) assumed a connection between serum lipid alterations and psychiatric disorders, including major depression, little research is available about the role of cholesterol metabolism in ADHD patients (Pinho et al. 2018). The formation of cholesteryl esters is central for the viscosity, fluidity and other physicochemical properties of biological membranes (Engelberg 1992). Animal experiments have suggested that a decrease in serum cholesterol may lead to lower serotonin in the brain resulting in a poorer impulse control (Engelberg 1992), which is a main symptom of ADHD. These findings demonstrate that alterations of lipid formation in neuronal membranes may be relevant for the pathogenesis of ADHD.

Using data from the nationwide and representative German Health Interview and Examination Survey for Children and Adolescents (Kinder- und Jugendgesundheitssurvey, KiGGS), we previously reported weak but statistically significant associations between ADHD symptoms and lower serum LDL cholesterol as well as higher triglyceride concentrations (Pinho et al. 2018). These results are in contrast to a small Turkish study conducted by Avcil (2018) which showed that boys with the clinical diagnosis of ADHD exhibited significantly lower values for total cholesterol, LDL and HDL compared to a control group but no alterations were found for triglyceride concentrations. Another study conducted by Irmisch et al. (2011) found significantly higher HDL values in ADHD patients compared to controls. These very heterogeneous and controversial results underline the fact that further research on this topic is necessary in order to gain a clearer picture of what could be expected from treatment/supplementation approaches. Specifically, the probable change of lipoproteins towards “normalization” during development into young adulthood and the predictive value of its early measurement would be highly informative in this respect. Hence, the aim of this post-hoc analysis was to examine the association between lipoproteins and ADHD with more stringent methods and to investigate whether children and adolescents with ADHD may display long-term changes in their peripherally measurable serum lipoprotein fractions. To this end, we included data from the second wave of the German KiGGS study which was conducted almost ten years after the baseline survey.

## Materials and methods

### KiGGS study population

This analysis is based on data from the public-use file of the longitudinal, nationwide and representative German KiGGS study. This survey was conducted by the Robert Koch Institute (RKI), Berlin, Germany (2018, also accesses Jan and Sep 2022), in three inquiry periods, namely baseline (2003–2006), Wave 1 (2009–2013) and Wave 2 (2014–2017) and financed by the German Federal Ministry of Health (Mauz et al. 2017). The aim of the study was to gather representative information about the general health status of children and adolescents aged up to 17 years in Germany by collecting data on social, physical and psychological parameters. For this post-hoc analysis, we only used data from baseline and Wave 2 since Wave 1 was exclusively conducted as a telephone-based interview and did not include any physical examination or laboratory measurements (Schlack et al. 2011). Baseline data were collected from questionnaires, physical examinations, computer-supported interviews by physicians and laboratory measurements were conducted at 167 sample points distributed all over Germany. The sample points were randomly selected and individuals were chosen from the official register of residents. Questionnaires were filled in by parents. Children aged 11 years or older received a self-rated questionnaire. In total, 17,641 participants were recruited for this prospective cohort-study (8985 boys and 8656 girls). The response rate was 66.6% (Kurth et al. 2008). For our analysis, the cohort included *n* = 10,960 children aged between 7 and 17 years as inclusion criteria. This age frame was chosen, since according to the Diagnostic and Statistical Manual of Mental Disorders (DSM-V), ADHD must be diagnosed before the age of 7 years (American Psychiatric Association 2013). Other variables, including sex and SES, were used to exclude individuals from matching (see “Statistical analysis”).

Data for the second follow-up survey, termed “Wave 2”, were collected between September 2014 and August 2017. Both, KiGGS baseline and KiGGS Wave 2 include an interview and an examination part (Mauz et al. 2017). In total, more than 10,500 young people from the original cohort sample (baseline) were enlisted again ten year later, meaning that almost two-thirds re-participated in the second follow-up. Wave 2 is separated into two parts: the follow-up of the baseline cohort (longitudinal data) and a newly enrolled group with children aged up to 17 years for cross-sectional analysis. The age-range of the follow-up cohort was 10 to 29 years. Particularly for older study participants, the drop-out rate was relatively high and their recruitment turned out to be more difficult due to their mobility away from their original sample point. Therefore, before starting Wave 2, the participants were separated into two groups depending on whether they still lived at the sample point or had moved away. The ones who had left the original location were only interviewed, and examination data, including information about blood test, were available only for 6465 subjects, equal to 36.6% of the baseline sample (Lange et al. 2018). Among these, a total number of *n* = 571 study participants were included in our study.

The ethics committee and Federal Office for the Protection of Data of Charitè/University Medical Center Berlin approved the baseline survey (No. 101/2000). KiGGS Wave 2 (No. 2275-2014) was checked for data protection by the Federal Commissioner for Data Protection and Freedom of Information in Germany and Hannover Medical School’s ethics committee with respect to the ethics (Lange et al. 2018; Ganjeh et al. 2021). To ensure the representativeness on a national level, the study was weighted by age, sex, residence in Western or Eastern Germany and nationality (Kurth et al. 2008). The KiGGS study was tested for its strengths as a cohort study using the Methodology Checklist 3 (Scottish Intercollegiate Guidelines Network (SIGN) 2004).

### Case definition of ADHD at baseline

Participants’ information on their putative ADHD diagnosis was collected from two sources: first, parents (for children up to 11 years) and the participants themselves (11 years and older) reported whether they had been diagnosed with ADHD by a physician or clinical psychologist by filling in the questionnaire at baseline. Secondly, the screening instrument Strengths and Difficulties Questionnaire (SDQ) was used for detection of children with ADHD symptoms independent of the diagnosis ADHD (Becker et al. 2015; Holtmann et al. 2011; Huss et al. 2008). Here, the hyperactivity-inattention (SDQ-H/I) sub-scale was used for assessment of suspected or potential ADHD with a predefined cut- off ≥ 7.

In total, study participants were classified into two groups: (1) the ADHD group, including study participants who had been given the diagnosis of ADHD (either by a medical doctor or a psychologist) before entering baseline investigation and/or had (at baseline assessment) a SDQ-H/I score of ≥ 7 on the hyperactivity-inattention subscale and (2) a control group with no clinical diagnosis of ADHD nor a SDQ-H/I score above the cut off.

Data about medication usage were also gathered using the questionnaire filled in by the parents or children during the computer assisted medical interview. First, medication usage during the last seven days was inquired into, in general (“Did your child intake any medication during the last seven days”). Afterwards it was further specified, e.g. “Who prescribed the drug”, “Why has it been prescribed”, “What is the name of the drug” etc. Afterwards the medication names were standardized using the ATC classification, for indication of the drug ICD 10 codes were used (Knopf 2007). Methylphenidate is the first line medicinal treatment for ADHD (Banaschewski et al. 2017b), we, therefore, considered study participants who took this drug or an equivalent preparation during the last seven days. In an extra analysis we further divided participants into the ADHD group users and non-users of methylphenidate. Here, we only included methylphenidate and no other stimulants such as atomoxetine, because it is part of the first line treatment. Secondly, methylphenidate was assumed to alter serum lipids (Charach et al. 2009) and we wanted to rule out this confounder. Information on medication usage relied on the parents’ questionnaire and on the self-questionnaire (14 years and older) (Robert Koch-Institut (RKI) and Bundeszentrale für gesundheitliche Aufklärung).

### Measurements of lipids

The measurement of lipids was conducted centrally in Berlin at the laboratories of the German “Robert-Koch-Institute” and the German Heart Centre, according to standardized protocols (Hölling et al. 2007). To assure and fulfil high quality requirements, external (inter-laboratory comparison) and internal quality control measures were conducted (Thierfelder et al. 2007). For practical reasons of data acquisition, the laboratory measurements were conducted at any time point during the day. It was not possible to consider any certain fasting time before the blood collection (Thierfelder et al. 2007). Venous blood samples were taken at mobile laboratories at the sample points and transported to Berlin within three days. The average time period was 18 h between sample taking and analysis. Prior to transportation, ETDA samples were stored at 4 °C. Serum samples were kept at an ambient temperature for 45 ± 15 min in Vacutainer gel tubes (Becton Dickinson), thereafter centrifuged, divided into aliquots and then stored at − 50 °C until further use. For all four lipid parameters (total cholesterol, HDL, LDL and triglycerides) serum samples were analysed enzymatically on a Hitachi 917 analyser (Roche, Mannheim). Results from lipid measurements are presented in mmol/l (Thierfelder et al. 2007; Pinho et al. 2018).

### Assessment of epidemiological data

For each study participant, questionnaires documented information about health status, health-related behaviors (e.g. smoking), living conditions, protective and risk factors and health care utilization (Robert Koch-Institut (RKI)). The computer-assisted personal interview collected detailed data on medication usage classifying them with the specific ATC (Anatomical Therapeutic Chemical) codes. Anthropometric data, including body height and weight, waist and hip circumferences, were measured using infantometers or stadiometers (Holtain Ltd., UK) and electronic scales (SECA, Ltd., Germany). This information was also used to calculate the body-mass-index (BMI) as weight in kilograms divided by height in metres squared (Kurth et al. 2008). To define the socioeconomic status (SES), a dimensional index was built including household income, education level and professional status. The basis for household income was the equivalent net income and for education level the international classification Comparative Analysis of Social Mobility in Industrial Nations (CASMIN). For professional status, the International Socio-Economic-Index of Occupational Status (ISEI) according to Ganzeboom and Treimann was used, always taking the maximum value given by the parents. The index ranges between 3.0 and 21.0 (Lampert et al. 2018).

### Statistical analysis

Laboratory measurements and demographic variables were analyzed in a descriptive manner. Categorical predictors are presented as percentages and continuous variables (e.g., age, BMI and heart rate and lipid parameters) are given as means and standard deviations. For a comparison between participants in the ADHD group and controls, chi-square tests were used for categorical variables and Student’s *t* tests for continuous variables. To improve the comparability of the two groups, propensity score matching was applied. This method estimates the group effect within observational datasets in order to minimize the selection bias by balancing the covariate distribution. Selected individuals appear similar with respect to the observed covariates while mainly differing in their classification to either the ADHD or control group (Rosenbaum and Rubin 1983). By creating a model that predicted the probability of being part of either the ADHD group or control group, a propensity score was generated for each individual and this score was then used for matching the subjects in the control group. In this study, we used the method of the nearest neighbor matching. Each participant in the ADHD group was assigned to an individual in the control group who showed the closest propensity score (i.e., the smallest distance). Data from non-selected controls were discarded and the results of the distribution of propensity scores before and after matching are presented in bar charts. As a sensitivity analysis, the potential lipid alterations caused by medication use was considered. Using analysis of variance (ANOVA), we compared the difference in lipoproteins levels between the three different groups, namely an ADHD group with medication usage (ADHD med) and one without medication usage (ADHD non-med) as well as controls. Given the absence of significant difference between participants in the ADHD group with and without medication usage, a single ADHD group will be used in the analyses. After creating a more comparable and homogenous sample of children in the ADHD and control groups (hereinafter termed as “matched sample”), the predictive value of lipid parameters on ADHD was tested using logistic regression models. Univariate logistic regression (i.e., unadjusted model) was used for testing whether lipid parameters (total cholesterol, LDL, HDL and triglycerides) predicted ADHD (i.e., individual’s group membership in the ADHD vs control group). Thereafter, multivariate logistic regressions (i.e., the adjusted models) were calculated on the four lipid parameters while controlling for demographic and somatic parameters. The “followed-and-matched sample” is the group of individuals who were included in the matched group at baseline and participated in the follow-up ten years later. This sub-group was analyzed descriptively including comparisons on measurements at baseline, follow-up and the differences between the two inquiry periods. In order to test the predictive value of the lipid parameters multivariate, logistic regression models were conducted in the followed-and-matched sample at (1) baseline to investigate the sensitivity of relationship due to the shrinkage of sample size and at (2) follow-up to search for differences in long-term changes. All statistical analyses were performed in R version 3.6.1 (R core team, 2019) and in all tests statistical significance was defined as *p* < 0.05.

## Results

### Characterization of the study cohort at baseline without matching

In total, 10,960 children of the KiGGS study aged between 7 and 17 were enrolled at baseline. Among them, *n* = 603 participants (5.5%) had been diagnosed with ADHD by a clinical psychologist or physician and *n* = 888 (8.1%) were suspected of having ADHD according to the SDQ-H/I score. Since some of the diagnosed participants showed also an elevated SDQ-H/I score, there was an overlap of *n* = 272 participants. Thus, both groups together (diagnosed and suspected ADHD cases) amounted to *n* = 1219, corresponding to 11.1% of all children. The remaining participants (*n* = 9741; 88.9%) were considered as control group. As described in the left panel of Table 1, participants in the defined ADHD group showed a mean SDQ-H/I score of 6.99 ± 1.90 before matching (6.16 for pre-diagnosed children and 7.93 for children with an elevated SDQ-score above the predefined cut-off), whereas this score was lower in the control group (2.54 ± 1.79). In our sample, 73.3% of the ADHD subgroup were male subjects and only 26.7% female (χ^2^ (1, *N* = 10,960) = 264.44, *p* < 0.001, Cohen’s ω = 0.156) confirming previous findings (Faraone et al. 2015). Children affected by ADHD were more likely to have lower SES than controls (*t* (10,651) = − 9.528, *p* < 0.001, Cohen’s *d* = 0.291), they were younger (*t* (10,958) = − 6.997, *p* < 0.001, Cohen’s *d* = 0.213) and had a lower BMI (*t* (10,908) = − 2.676, *p* < 0.001, Cohen’s *d* = 0.082).

**Table 1:** Characterization of participants in the ADHD and control group at KiGGS baseline (before and after matching)

	Before matching				After matching			
	Participants in the ADHD group (*n* = 1219)	Participants in the control group (*n* = 9741)	*p *value	Effect size	Participants in the ADHD group (*n* = 1190)	Participants in the control group (*n* = 1190)	*p *value	Effect size
Age (years)	11.25 ± 2.91	11.92 ± 3.13	< 0.001	0.213	11.26 ± 2.92	11.27 ± 3.07	0.891	0.006
Sex (%). female	26.74	51.48	< 0.001	0.156	26.81	26.64	0.963	0.002
SDQ-H/I-score	6.99 ± 1.90	2.54 ± 1.79	< 0.001	2.474	6.97 ± 1.91	2.80 ± 1.81	< 0.001	0.041
SES Index	10.32 ± 4.16	11.58 ± 4.33	< 0.001	0.291	10.33 ± 4.17	10.35 ± 4.17	0.914	0.004
SES category (%)			< 0.001	0.092			0.907	0.009
Low	37.17	26.13			37.23	36.97		
Medium	46.61	47.62			46.47	46.05		
High	16.23	26.25			16.30	16.97		
BMI (kg/m^2^)	19.28 ± 4.20	19.61 ± 4.00	0.007	0.082	19.26 ± 4.19	19.31 ± 3.94	0.800	0.010
BMI category (%)			< 0.001	0.045			0.809	0.020
Underweight (BMI < 18.5)	50.53	44.09			51.01	50.00		
Normal (18.5 ≤ BMI < 25.0)	38.97	45.92			39.08	40.67		
Overweight (25.0 ≤ BMI < 30.0)	8.04	7.31			8.15	7.90		
Obese (BMI > 30.0)	2.46	2.68			1.76	1.43		
Heart rate (bpm)	78.61 ± 11.73	78.47 ± 11.78	0.683	0.012	78.54 ± 11.61	78.26 ± 11.63	0.565	0.024
Total cholesterol (mmol/l)	4.25 ± 0.71	4.27 ± 0.74	0.399	0.027	4.25 ± 0.70	4.24 ± 0.71	0.790	0.014
LDL (mmol/l)	2.39 ± 0.65	2.42 ± 0.66	0.115	0.051	2.39 ± 0.64	2.40 ± 0.64	0.602	0.012
HDL (mmol/l)	1.50 ± 0.35	1.50 ± 0.34	0.444	0.025	1.51 ± 0.35	1.50 ± 0.35	0.557	0.006
Triglycerides (mmol/l)	1.27 ± 0.76	1.23 ± 0.73	0.151	0.046	1.26 ± 0.75	1.23 ± 0.74	0.323	0.023

Annotations: age (years); sex (percentage of female participants)

For effect sizes, Cohen’s *ω* for sex, SES category (%) und BMI category (%) was used, for all others Cohen’s *d*

*SDQ-H/I* strengths and difficulties questionnaire subscale hyperactivity/inattention, *SES* socioeconomic status, *BMI* body mass index (kg/m^2^), heart rate (*bpm* beats per minutes), total cholesterol (mmol/l), *LDL* low-density lipoprotein (mmol/l), *HDL*high-density lipoprotein (mmol/l); triglycerides in (mmol/l)

### Distribution of the propensity score matching

**Fig. 1:**
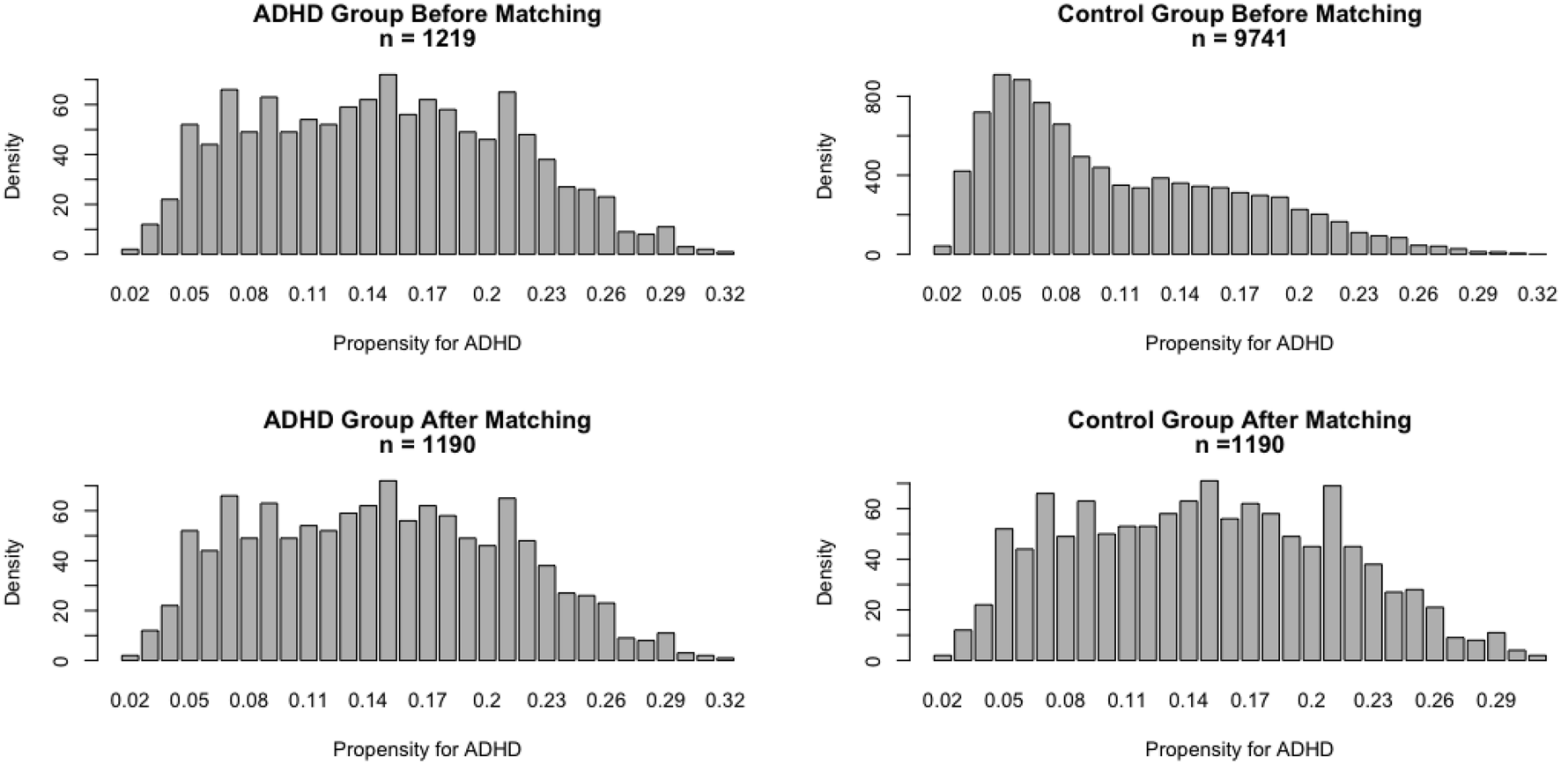
Distribution of propensity scores for ADHD before and after matching

For propensity score matching, the nearest neighbour matching technique was used. The distribution before and after propensity score matching is depicted in Fig. 1. This matching technique was used to improve comparability and to correct the confounders in the ADHD and control groups on observed interfering factors. The upper panel of Fig. 1, presenting participants’ propensity scores of ADHD risk based on indicators (i.e., age, sex ratio, SES, BMI and heart rate), showed almost a normal distribution for the ADHD group (*n* = 1219). The distribution within the control group (*n* = 9741) is more skewed. The panel below illustrates the distribution after propensity score matching, demonstrating that the procedure resulted in a more normal distribution after matching, closer to the one observed for the ADHD group. In total, 1190 participants were included in the ADHD group and an equal number in the controls, having the closest propensity to ADHD risks. Consequently, participants of both groups presented similar characteristics in age (t (2378) = − 0.137, *p* = 0.891, Cohens *d* = 0.006), sex ratio (χ^2^ (1, *N* = 2380) = 0.002, *p* = 0.963, Cohens *ω* = 0.002), SES (*t* (2378) = − 0.108, *p* = 0.914, Cohens *d* = 0.004) and BMI (*t* (2378) = − 0.253, *p* = 0.800, Cohens *d* = 0.010). Details are shown in the right-hand panel of Table 1.

Valid information about the intake of medication for ADHD during the study period was available only for a small group and thus was not included in this analysis as a confounder. However, since methylphenidate is the first line drug in ADHD treatment and was suspected of causing hypocholesterolemic and hypotriglyceridemic effects (Charach et al. 2009), we analysed the difference in lipoprotein levels between participants in the ADHD group with and without methylphenidate intake in a sensitivity analysis (see appendix). Findings suggest there were no significant effects of methylphenidate intake on the lipids.

### Lipid parameters in KiGGS baseline cohort before matching

In the KiGGS baseline cohort, there were no significant differences between the ADHD group and controls for all four lipid parameters tested (total cholesterol, t (10,101) = − 0.844, *p* = 0.399, Cohens *d* = 0.027; LDL *t*(10,103) = − 1.578, *p* = 0.115, Cohens *d* = 0.051; HDL *t*(10,102) = 0.766, *p* = 0.444, Cohens *d* = 0.025; triglycerides *t*(10,103) = 1.437, *p* = 0.151, Cohens *d* = 0.046, Table 1). In Table 2 (upper panel), results of unadjusted, univariate logistic regression analysis are presented. Before matching, important confounders, including age, sex, SES and BMI were all significantly associated with ADHD.

**Table 2:** Results from unadjusted and adjusted logistic regression models with ADHD as dependent variable in the KiGGS baseline cohort (before and after matching). Unadjusted models used univariate logistic regression

	Before matching (10,960)				After matching (*n* = 2380)			
	Exp(*ß*)	95%-CI	Wald	*p *value	Exp(*ß*)	95%-CI	Wald	*p* value
Unadjusted models								
Age	0.933	0.915–0.952	− 6.953	< 0.001	0.998	0.972–1.025	− 0.137	0.891
Sex	0.344	0.301–0.392	− 15.736	< 0.001	1.009	0.841–1.210	0.093	0.926
SES	0.932	0.918–0.946	− 9.423	< 0.001	0.999	0.980–1.018	− 0.108	0.914
BMI	0.979	0.964–0.994	− 2.674	0.007	0.997	0.978–1.017	− 0.253	0.800
Heart rate	1.001	0.996–1.006	0.408	0.683	1.002	0.995–1.009	0.575	0.565
Cholesterol	0.964	0.884–1.049	− 0.844	0.399	1.016	0.902–1.146	0.267	0.789
LDL	0.925	0.839–1.018	− 1.578	0.115	0.965	0.846–1.102	− 0.521	0.602
HDL	1.074	0.894–1.287	0.766	0.444	1.074	0.846–1.364	0.588	0.557
Triglycerides	1.062	0.977–1.150	1.437	0.151	1.059	0.946–1.187	0.989	0.323
Adjusted model for cholesterol								
Age	0.935	0.911–0.960	− 5.039	0.000	1.007	0.973–1.043	0.420	0.674
Sex	0.347	0.300–0.400	− 14.464	0.000	1.013	0.836–1.228	0.133	0.894
SES	0.931	0.917–0.946	− 8.779	0.000	0.998	0.978–1.019	− 0.206	0.837
BMI	0.998	0.979–1.017	− 0.199	0.843	0.991	0.967–1.015	− 0.754	0.451
Heart rate	0.998	0.992–1.004	− 0.646	0.518	1.002	0.995–1.010	0.592	0.554
Cholesterol	0.999	0.911–1.094	− 0.027	0.979	1.016	0.900–1.149	0.261	0.794
Adjusted model for LDL								
Age	0.934	0.910–0.959	− 5.139	0.000	1.005	0.971–1.041	0.305	0.760
Sex (f)	0.349	0.302–0.402	− 14.411	0.000	1.020	0.841–1.236	0.197	0.844
SES	0.931	0.917–0.946	− 8.768	0.000	0.998	0.978–1.019	− 0.188	0.851
BMI	0.999	0.980–1.018	− 0.130	0.897	0.992	0.968–1.016	− 0.680	0.496
Heart rate	0.998	0.992–1.004	− 0.621	0.535	1.002	0.995–1.010	0.610	0.542
LDL	0.967	0.872–1.071	− 0.635	0.525	0.966	0.844–1.106	− 0.500	0.617
Adjusted model for HDL								
Age	0.935	0.911–0.960	− 5.079	0.000	1.007	0.973 – 1.042	0.390	0.696
Sex	0.346	0.300–0.399	− 14.554	0.000	1.014	0.837 – 1.229	0.146	0.884
SES	0.931	0.916–0.946	− 8.787	0.000	0.998	0.978 – 1.019	− 0.205	0.838
BMI	1.001	0.981–1.020	0.050	0.960	0.992	0.968 – 1.018	− 0.590	0.556
Heart rate	0.998	0.992–1.004	− 0.633	0.527	1.002	0.995 – 1.010	0.608	0.543
HDL	1.095	0.899–1.331	0.904	0.366	1.053	0.817 – 1.358	0.400	0.689
Adjusted model for triglycerides								
Age	0.936	0.912–0.960	− 5.041	0.000	1.008	0.974–1.043	0.450	0.653
Sex	0.348	0.301–0.401	− 14.490	0.000	1.018	0.840–1.233	0.180	0.857
SES	0.931	0.916–0.946	− 8.795	0.000	0.998	0.977–1.018	− 0.227	0.821
BMI	0.996	0.976–1.016	− 0.400	0.689	0.987	0.963–1.012	− 0.999	0.318
Heart rate	0.998	0.992–1.004	− 0.684	0.494	1.002	0.994–1.010	0.525	0.600
Triglycerides	1.038	0.948–1.133	0.820	0.412	1.073	0.954–1.207	1.169	0.243

Adjusted models used multivariate logistics

Annotations: 95%-CI = 95% confidence interval; age (years)

*SES* socioeconomic status, *BMI* body mass index (kg/m^2^), heart rate (*bpm* beats per minutes), total cholesterol (mmol/l), *LDL* low-density lipoprotein (mmol/l), *HDL* high-density lipoprotein (mmol/l), triglycerides in (mmol/l)

In the multivariate logistic models, the associations between lipid parameters and ADHD were tested with the confounders each in separate models (Table 2). Only age, sex and SES were still significantly associated with ADHD, neither BMI nor the lipid variable showed a significant association with ADHD: total cholesterol (Exp(*ß*) = 0.999, 95%-CI 0.911–1.094, *p* = 0.979), LDL (Exp(*ß*) = 0.967, 95%-CI 0.872–1.071, *p* = 0.525), HDL (Exp(*ß*) = 1.095, 95%-CI 0.899–1.331, *p* = 0.366) and triglycerides (Exp(*ß*) = 1.038, 95%-CI 0.948–1.133, *p* = 0.412).

### Lipid parameters in KiGGS baseline cohort after matching

For the matched sample including 2380 participants, the same analysis process was performed again with ADHD as dependent variable. As individuals were matched for their age, sex, SES and BMI, these factors were no longer associated with ADHD in the matched sample. As shown in the right-hand panel of Table 2, there were no statistically significant associations between ADHD and either of the serum lipid concentrations: total cholesterol *t*(2157) = 0.267, *p* = 0.789, Cohen’s *d* = 0.014, LDL *t*(2,157) = − 0.521, *p* = 0.602, Cohen’s *d* = 0.012; HDL *t*(2157) = 0.588, *p* = 0.557, Cohen’s *d* = 0.006 and triglycerides *t*(2157) = 0.989, *p* = 0.323, Cohen’s *d* = 0.023. The corresponding adjusted, multivariate models confirmed these results for total cholesterol (Exp(*ß*) = 1.016, 95%-CI 0.900–1.149, *p* = 0.794), LDL (Exp(*ß*) = 0.966, 95%-CI 0.844–1.106, *p* = 0.617), HDL (Exp(*ß*) = 1.053, 95%-CI 0.817–1.358, *p* = 0.689) and triglycerides (Exp(*ß*) = 1.073, 95%-CI 0.954–1.207, *p* = 0.243).

### Characterization of the study cohort at follow-up after matching

For a follow-up 10 years later, 6,044 participants from the baseline were enlisted again for laboratory measurements and physical examinations (Kurth 2018). A total number of *n *= 571 fulfilled the criteria for inclusion in our study cohort, representing roughly a quarter of the baseline study cohort. Among them, *n* = 258 subjects belonged to the ADHD group and *n *= 313 to the control group according to their group membership at baseline. The matching between the ADHD group and control group remained effective, as the level of confounding variables remained similar between groups: age (*t* (569) = 0.146, *p* = 0.884, Cohen’s *d* = 0.012), SES (*t* (569) = 0.638, *p* = 0.524, Cohen’s *d* = 0.054), BMI (*t* (567) = 2.533, *p* = 0.012, Cohen’s *d* = 0.170) and heart rate (t(569) = 1.607, *p* = 0.109, Cohen’s *d* = 0.135). In univariate analysis, there were no significant differences between the ADHD and control groups with respect to lipid parameters (for details see Table 3). The relationships between single lipid parameters and ADHD were than analysed using multivariate logistic regression models with ADHD as dependent variable. Total cholesterol, LDL, HDL and triglycerides were set as independent variables in separated models adjusted for age, sex, BMI, SES and heart rate. Again after controlling for these confounders, no significant associations were observed between the lipid variables and ADHD: total cholesterol (Exp(*ß*) = 0.972, 95%-CI 0.789–1.182, *p* = 0.775), LDL (Exp(*ß*) = 0.998, 95%-CI 0.784–1.270, *p* = 0.990), HDL (Exp(*ß*) = 0.928, 95%-CI 0.480–1.789, *p* = 0.823) and triglycerides (Exp(*ß*) = 1.047, 95%-CI 0.863–1.268, *p* = 0.636). Details are shown in Table 4 with results for baseline data in the left-hand panel and follow-up on the right.

**Table 3:** Characterization of matched participants in the ADHD and control group at KiGGS follow-up

	Participants in the ADHD group (*n* = 258)	Participants in the control group (*n* = 313)	*p* value	Effect size
Measurement at baseline				
Age (years)	10.65 ± 2.94	10.62 ± 3.01	0.890	0.012
Sex (%, f)	28.29	24.28	0.322	0.045
SDQ-H/I	6.95 ± 1.95	2.74 ± 1.81	< 0.001	0.366
SES Index	10.90 ± 3.97	10.69 ± 4.08	0.524	0.054
SES category (%)			0.444	0.053
Low	29.84	34.82		
Middle	50.39	46.33		
High	19.77	18.85		
BMI (kg/m^2^)	18.70 ± 4.17	18.50 ± 3.57	0.535	0.052
BMI category (%)			0.443	0.069
Underweight	60.85	59.74		
Normal	31.40	33.55		
Overweight	6.20	6.39		
Obese	1.55	0.32		
Heart rate (bpm)	79.69 ± 11.39	78.79 ± 10.93	0.339	0.080
Cholesterol (mmol/l)	4.33 ± 0.76	4.24 ± 0.68	0.162	0.060
LDL (mmol/l)	2.44 ± 0.66	2.40 ± 0.61	0.453	0.033
HDL (mmol/l)	1.53 ± 0.36	1.51 ± 0.34	0.487	0.001
Triglycerides (mmol/l)	1.29 ± 0.75	1.22 ± 0.75	0.274	0.053
Measurement at follow-up				
Age (years)	21.57 ± 2.93	21.54 ± 3.06	0.884	0.012
BMI (kg/m^2^)	24.93 ± 5.47	23.92 ± 4.03	0.012	0.170
BMI category (%)			0.015	0.135
Underweight	4.26	5.11		
Normal	56.59	60.06		
Overweight	22.09	26.52		
Obese	17.05	8.31		
Heart rate (bpm)	75.08 ± 13.06	73.41 ± 11.77	0.109	0.135
Cholesterol (mmol/l)	4.57 ± 0.94	4.52 ± 0.91	0.471	0.061
LDL (mmol/l)	2.61 ± 0.75	2.55 ± 0.72	0.305	0.086
HDL (mmol/l)	1.35 ± 0.30	1.36 ± 0.31	0.570	0.048
Triglycerides (mmol/l)	1.43 ± 0.99	1.32 ± 0.86	0.170	0.115
Baseline vs. follow-up change				
Age (years)	10.92 ± 0.42	10.92 ± 0.40	0.946	0.006
BMI (kg/m^2^)	6.23 ± 3.97	5.43 ± 3.25	0.008	0.135
Heart rate (bpm)	− 4.61 ± 13.15	− 5.38 ± 13.26	0.486	0.059
Cholesterol (mmol/l)	0.26 ± 0.89	0.29 ± 0.82	0.740	0.178
LDL (mmol/l)	0.18 ± 0.68	0.15 ± 0.61	0.671	0.161
HDL (mmol/l)	− 0.18 ± 0.35	− 0.14 ± 0.34	0.300	0.010
Triglycerides (mmol/l)	0.16 ± 1.11	0.13 ± 1.11	0.712	0.082

Annotations: age (years); sex (percentage of female participants)

For effect strengths, Cohen’s *ω* for sex. SES category (%) und BMI category (%) was used, for all others Cohen’s *d*

*SDQ-H/I* strengths and difficulties questionnaire subscale hyperactivity/inattention, *SES* socioeconomic status, *BMI* body mass index (kg/m^2^), heart rate (*bpm* beats per minutes), total cholesterol (mmol/l), *LDL* low-density lipoprotein (mmol/l), *HDL* high-density lipoprotein (mmol/l); triglycerides in (mmol/l)

**Table 4:** Results from adjusted logistic regression models with ADHD as dependent variable in the matched sample retained at KiGGS follow-up

	Lipid parameters at baseline				Lipid parameter at follow-up			
	Exp(*ß*)	95% CI	Wald	*p *value	Exp(*ß*)	95% CI	Wald	*p*-value
Adjusted model for cholesterol								
Age	1.003	0.935–1.076	0.093	0.926	1.002	0.945–1.061	0.058	0.954
Sex	1.157	0.776–1.726	0.717	0.473	1.247	0.833–1.867	1.072	0.284
SES	1.016	0.973–1.061	0.739	0.460	1.024	0.982–1.068	1.108	0.268
BMI	1.016	0.964–1.070	0.588	0.556	1.052	1.014–1.093	2.669	0.008
Heart rate	1.009	0.993–1.026	1.134	0.257	1.011	0.997–1.025	1.600	0.110
Total cholesterol	1.163	0.913–1.486	1.215	0.224	0.972	0.798–1.182	− 0.286	0.775
Adjusted model for LDL								
Age	1.001	0.933–1.073	0.027	0.978	1.000	0.945–1.059	0.014	0.989
Sex	1.175	0.788–1.751	0.792	0.429	1.227	0.829–1.817	1.026	0.305
SES	1.017	0.974–1.062	0.780	0.435	1.024	0.982–1.068	1.093	0.274
BMI	1.017	0.965–1.071	0.622	0.534	1.051	1.013–1.093	2.583	0.010
Heart rate	1.009	0.993–1.026	1.146	0.252	1.011	0.997–1.025	1.569	0.117
LDL	1.080	0.821–1.422	0.551	0.581	0.998	0.784–1.270	− 0.012	0.990
Adjusted model for HDL								
Age	0.998	0.931–1.070	− 0.048	0.961	1.001	0.945–1.061	0.050	0.960
Sex	1.200	0.806–1.785	0.899	0.369	1.257	0.808–1.956	1.015	0.310
SES	1.017	0.975–1.062	0.788	0.431	1.024	0.982–1.068	1.097	0.272
BMI	1.025	0.972–1.082	0.912	0.362	1.050	1.010–1.092	2.454	0.014
Heart rate	1.010	0.994–1.026	1.231	0.218	1.011	0.997–1.025	1.555	0.120
HDL	1.307	0.783–2.188	1.023	0.306	0.928	0.480–1.789	− 0.223	0.823
Adjusted model for triglycerides								
Age	1.000	0.933–1.071	− 0.008	0.993	1.000	0.945–1.059	0.008	0.994
Sex	1.184	0.796–1.762	0.835	0.403	1.236	0.837–1.826	1.069	0.285
SES	1.017	0.974–1.062	0.765	0.444	1.023	0.981–1.067	1.060	0.289
BMI	1.013	0.961–1.069	0.493	0.622	1.049	1.011–1.090	2.476	0.013
Heart rate	1.009	0.993–1.025	1.052	0.293	1.011	0.997–1.025	1.476	0.140
Triglycerides	1.099	0.867–1.407	0.775	0.438	1.047	0.863–1.268	0.473	0.636

Adjusted models used multivariate logistic regression with total cholesterol, LDL, HDL and triglycerides at baseline

Annotations: 95% CI 95% confidence interval; age (years)

*SES* socioeconomic status, *BMI* body mass index (kg/m^2^), heart rate (*bpm* beats per minutes); total cholesterol (mmol/l); *LDL* low-density lipoprotein (mmol/l), *HDL* high-density lipoprotein (mmol/l), triglycerides in (mmol/l)

### Changes between baseline and follow-up

The lower part of Table 3 illustrates the difference between baseline and follow-up. Except for the BMI, all other measurements remained stable over time. For the BMI, it emerges that during baseline most of the participants showed values that ranged around normal- and underweight (≤ 24.9). At follow-up 10 years later, when the majority of the participants were already in their early adulthood, they had significantly higher values for BMI. More than one third of the participants were classified as overweight or even obese (BMI ≥ 25).

Overweight and obesity could have an impact on lipid parameters (Deeb et al. 2018; Kase et al. 2021). Our additional analysis supports the assumption that there is an association between BMI and lipid levels at follow-up, as shown in the Supplementary Table 3. However, even in participants with overweight or obesity, we could not find any association between ADHD and lipid levels, as shown in the Supplementary Table 4.

## Discussion

This multi-cross-sectional investigation of German children and adolescents aged between 7 and 17 years at baseline aimed to study long-term changes in serum lipid concentrations between subjects with and without ADHD. The KiGGS study has the unique opportunity of being a large, representative baseline cohort and a somewhat smaller longitudinal follow-up study. More than 1200 children and adolescents were assessed for their lipid profile providing information about changes to young adulthood over a ten-year follow-up time. Unlike previous studies (Irmisch et al. 2011; Avcil 2018; Pinho et al. 2018), this analysis was unable to find significant relationships between the lipid profile and ADHD, neither for cross-sectional nor longitudinal comparison in both univariate analysis and multivariate models.

By using the stringent method of propensity score matching, we ensured the comparability between the ADHD and control group and, in addition, ruled out potential confounding by age, sex, SES, BMI and heart rate. Both before and after matching, we found no significant associations between ADHD and lipid parameters. The lack of any statistically significant relationships between ADHD and controls with the regard to lipid measurements, as demonstrated in our analysis, may be explained by the broader age group studied, as well as differences in inclusion criteria for ADHD matching processes, weighting strategies, and sample sizes.

With the use of propensity score matching, individuals received a very similar partner with respect to their socio-demographic and bodily characteristics. One important confounder, describing the participants’ physical constitution, is the BMI. The influence of BMI on lipid profile has been discussed widely in the literature. A study from the Freiburg Intervention Trial for Obese Children (FITOC) showed that nearly 50% of obese children had some kind of dyslipidaemia (Korsten-Reck et al. 2008) and also a small study in the Middle East came to similar results (Deeb et al. 2018). The long-term changes in our study showed that the weight indicated by BMI increased significantly over the years. At baseline, most children were normal- or underweight, whereas at follow-up (i.e. about 10 years later) most of these subjects were overweight or obese. This is consistent with the study of Kase et al. (2021), who interpreted the findings as a common genetic background. Although we demonstrated an association between BMI and lipid levels at follow-up, we did not find any association between ADHD and lipid levels, even in participants with overweight or obesity. However, due to our study design, no firm conclusions can yet be drawn about the trivalent association between increased BMI and ADHD and lipid parameters.

The negative findings for lipoproteins and ADHD remained stable over time. The non-significant results, presented here in this broad age group, do not rule out that alterations in serum lipoprotein concentrations are unrelated to the diagnosis of ADHD at all. Since, in a preliminary study, Charach et al. (2009) assumed positive hypocholesterolemic and hypotriglyceridemic effects for methylphenidate we particularly tried to test for any influence of this drug on peripheral lipid parameters. The results evidenced negative findings and suggested that the role of lipoproteins in ADHD seems to be less important than assumed so far.

Some recent work by Meijer et al. (2020) examined differences in epigenome-wide methylation for genes involved in cholesterol signalling between ADHD persisters, remitters and healthy controls. They reported associations between ADHD persistence and hypermethylated regions in the *APOB* and *LPAR5* genes in peripheral whole blood samples. Although the authors reason that their peripheral findings might also hold for brain-related processes, they noted that brain lipoproteins are exclusively produced in the brain and thus the results of their pilot study should be interpreted with caution (Meijer et al. 2020). Specifically, it remains unclear whether or not this may be related to clinical parameters of lipoproteins as measured in our study.

### Strengths and limitations

The data collection of the large-scale, epidemiological KiGGS study was prepared and conducted very stringently and results revealed a high level of representative accuracy on a national level and a high quality control (Kurth 2007). Moreover, characteristics of individuals with and without ADHD were adjusted by application of a strict matching process. Since data from two points were available, information about long-term changes could be analysed. This provided a stable and reliable basis for our analysis. Using propensity score matching, we increased the quality of the comparisons between children with and without ADHD by reducing the influence of potential confounders. Some limitations should be considered when interpreting our results.

As a major limitation participants did not fast before the sample taking. Although studies revealed that lipid results of fasting individuals have the same prognostic value as non-fasting ones, it still seems that triglycerides and LDL are altered after food intake (Visseren et al. 2021). Hence, without any information about fasting the results must be interpreted with great caution.

As already mentioned above, the medication usage and dietary habits during and before examination were not detailed and valid enough to utilize them for our analyses of the complete cohort. Furthermore, ADHD diagnosis was collected by trained medical personnel but also relied on parents’ report attendance with a low overlap of diagnosed and suspected ADHD cases (Kurth et al. 2008). In our cohort, previously described effects of methylphenidate could not be reproduced. These results are limited by the fact that data did not provide any information about the duration of medication intake. Moreover, it was just one measurement at one time point and we did not have any lipid values before starting the methylphenidate treatment. We were only able to conduct a limited analysis on the influence of medication intake, namely methylphenidate and results (in the appendix) must be interpreted with caution. The role of polyunsaturated fatty acids (PUFAs) was not part of the laboratory measurements in the KiGGS study and thus could not be included in our study (Thierfelder et al. 2007). Future research is needed to further clarify the role of PUFAs, as evidence linking ADHD to altered PUFA level is inconsistent or low in credibility (Chang et al. 2018; Händel et al. 2021; Gao et al. 2022). Finally, due to a lack of information on categorical mental health problems other than ADHD, we could not rule out the potential role of other associated psychiatric disorders.

## Conclusion

In a large representative sample of German children, we did not find evidence that peripheral lipid parameters are associated with ADHD, neither cross-sectionally when investigating adolescents and young adults, nor longitudinally during the development from adolescence into young adulthood. Hence, at the moment, the hypothesis of long-term lipoprotein alterations in ADHD cannot be supported by our epidemiological data. As this analysis focusses on peripheral alterations, we cannot draw any conclusions about biochemical or genetic mechanisms concerning the cholesterol metabolism in the brain. Since other studies have supported the hypothesis that the lipid metabolism may be relevant for neurodevelopmental diseases, further research at both, the molecular and clinical level, is necessary to clarify the exact role of serum lipids in the pathophysiology of ADHD.

## Supplementary Information

Below is the link to the electronic supplementary material.

Supplementary file1 (DOCX 141 KB)

## Data Availability

The data that support the findings of this study are available from Robert-Koch Institute (Berlin) but restrictions apply to the availability of these data, which were used under license for the current study, and so are not publicly available. Data are, however, available from the authors upon reasonable request and with permission of Robert-Koch Institute, Berlin.
