# Supplementary material for: Long-term changes in serum levels of lipoproteins in children and adolescents with attention-deficit/hyperactivity disorder (ADHD)
Source: J Neural Transm (Vienna). 2023 Feb 24;130(4):597–609. doi: 10.1007/s00702-022-02583-5 (PMC10050056; doi:10.1007/s00702-022-02583-5)
Supplement: Supplementary file 1 — Supplementary file1 (DOCX 141 KB) [file 702_2022_2583_MOESM1_ESM.docx]

**APPENDIX**

**Characteristics of the subgroup ADHD with Methylphenidate intake**

To control for lipid alterations caused by medication use a further subgroup “ADHD with Methylphenidate use” was analysed. This subgroup was extracted out of the matched baseline sample (ADHD prediagnosed or SDQ-H/I score of ≥ 7 on the hyperactivity-inattention subscale and controls). Data about the medication use was gathered using the questionnaire filled in by the parents or children during the computer assisted medical interview. First, medication use during the last seven days was inquired in general (“Did your child intake any medication during the last seven days”). Afterwards it was further specified, e.g. “who prescribed the drug”, “Why has it been prescribed”, “What is the name of the drug” etc. Afterwards the medication names were standardized using the ATC classification, for indication of the drug ICD 10 codes were used (Knopf 2007). Since Methylphenidate (MPH) is the first line medicinal treatment for ADHD (Banaschewski et al. 2017) we only included study participants who took MPH or an equivalent preparation during the last seven days. Here, we only included Methylphenidate and no other stimulants such as atomoxetine for two considerations. Firstly, we focused on the first line treatment. Secondly, MPH was assumed to alternate the serum lipids (Charach et al. 2009) and we wanted to rule out this confounder. Information about the medication intake relied on parents’ questionnaire and on childrens’ questionnaire (14 years and older) (Robert Koch-Institut (RKI) and Bundeszentrale für gesundheitliche Aufklärung). We divided the sample into three different groups, namely ADHD group with medication use “ADHD med”, ADHD group without medication use “ADHD non-med” and controls using an analysis of variance (ANOVA). Because of the small sample size of probands with MPH use, data could not be included as probable confounders in the general analysis; instead, a separate group comparison was done.

**Results of the study cohort “ADHD with Methylphenidate intake” at baseline**

In total only n = 138 of the matched baseline sample (n = 1,190) had valid data about MPH intake. This is equal to 11,6%. The remaining group “ADHD without MPH” consisted of n = 1,052. These participants did not intake any medication for ADHD or used another medication. The control group n = 1,190 was equal to the original sample and should not be described in this extra analysis. The percentage of female participants in this sample was only 13.77%, sex ratio (χ2 (2, N = 13.561, p = 0.001, Cohens ω = 0.075). Children with drug intake were more likely to have a medium SES and they presented more often a lower BMI than children without MPH intake (for details see Table  1).

We found no significant difference in lipid levels between individuals in the ADHD group with and without MPH intake: Total cholesterol t(140,36) =0.391, p = 0.697, Cohens d = 0.045, LDL t(140,81) = 0.450, p = 0.653, Cohens d = 0.051; HDL t(142,85) = 1.685, p = 0.094, Cohens d = 0.185; triglycerides t(152,4) = -1.448, p = 0.150, Cohens d = 0.139 Table 1).

**Results of the study cohort “ADHD with Methylphenidate intake” at follow-up**

In the matched follow-up sample data about MPH intake was available only for n = 26 participants being equal to 10,1% of the original sample (n = 258 ADHD complete). Also, in this analysis it revealed that girls are less likely to intake MPH since only 11.54% of the “ADHD with medication use at follow-up” were female compared to the group without MPH (30.17 % female participants). Moreover, it arised that the major part of participants with drug intake presented a medium or high SES. Age, BMI and heart rate showed similar percentual distribution. Here, also no significant difference in lipid levels between the groups appeared, for details see table 2.

**Lipid profile corrected for BMI**

In order to rule out a possible confounding between lipid parameters and BMI since a connection was suggested elsewhere (Deeb et al. 2018; Korsten-Reck et al. 2008; Kase et al. 2021), we did another calculation with a post hoc power analysis. Therefore, we divided the ADHD group into two subgroubs: (1) BMI underweight or normal and (2) BMI overweight or obese at follow-up. Neither for the subgroup under-/normal weight nor for the second group a significant association could be found. For details, please see table 3 and 4.

**Power analysis**

Moreover we did an additional power analysis. With the baseline original sample (nADHD = 1219, ncontrol = 9741), we have 99% power to detect a small effect of Cohen’s d = 0.2. With the baseline matched sample (nADHD = 1190, ncontrol = 1190), we have 99% power to detect a small effect of Cohen’s d = 0.2. With the follow-up sample (nADHD = 258, ncontrol = 313), we have 66% power to detect a small effect of Cohen’s d = 0.2.

**Discussion:**

Methylphenidate was suspected to alter serum lipid levels. Charach et al. (2009) analysed the serum lipids of 42 patients before starting the treatment with MPH and 3 month afterwards. Here, they found that MPH had a lowering effect on LDL, cholesterol and triglycerides. In our cohort these results could not be reproduced. Our results are limited by the fact that we analysed the lipid values only at one time point without having any value before starting the treatment. Anyhow, there was no significant effect between ADHD and lipids, even after controlling for MPH.

Table 1: Characterization of participants in the subgroups ADHD with and without Methylphenidate intake at KiGGS baseline after matching.

|  | **ADHD group (n = 1,190)**  **baseline after matching** | | | | |
| --- | --- | --- | --- | --- | --- |
|  | Participants with MPH (n = 138) | Participants without MPH (n = 1,052) | p-value | Effect size |  |
| Age | 11.09±2.54 | 11.28±2.97 | 0.411 | 0.113 |  |
| Sex (%), female | 13.77 | 28.52 | <0.001 | 0.10.7 |  |
| SDQ-H/I-value | 6.99±2.07 | 6.97±1.88 | 0.905 | 0.146 |  |
| SES Index | 11.20±4.18 | 10.22±4.15 | 0.010 | 0.157 |  |
| SES category (%) |  |  | 0.010 | 0.088 |  |
| Low | 26.09 | 38.69 |  |  |  |
| Medium | 52.17 | 45.72 |  |  |  |
| High | 21.74 | 15.59 |  |  |  |
| BMI | 18.71±4.07 | 19.34±4.20 | 0.094 | 0.225 |  |
| BMI category (%) |  |  | 0.391 | 0.050 |  |
| Underweight (BMI < 18.5) | 57.25 | 50.19 |  |  |  |
| Normal (18.5 ≤ BMI < 25.0) | 32.61 | 39.92 |  |  |  |
| Overweight (25.0 ≤ BMI < 30.0) | 8.70 | 8.08 |  |  |  |
| Obese (BMI > 30.0) | 1.45 | 1.81 |  |  |  |
| Heart rate (bpm) | 80.59±12.54 | 78.27±11.47 | 0.040 | 0.157 |  |
| Total cholesterol (mmol/l) | 4.28±0.85 | 4.24±0.68 | 0.697 | 0.045 |  |
| LDL (mmol/l) | 2.42±0.77 | 2.38±0.62 | 0.653 | 0.051 |  |
| HDL (mmol/l) | 1.56±0.41 | 1.50±0.35 | 0.094 | 0.185 |  |
| Triglycerides (mmol/l) | 1.17±0.75 | 1.27±0.75 | 0.150 | 0.139 |  |

Annotations: age (years); sex (percentage of female participants); SDQ-H/I=Strengths and Difficulties Questionnaire Subscale Hyperactivity/Inattention; SES=socioeconomic status; BMI=Body Mass Index (kg/m²). Heart rate (bpm = beats per minutes); Total Cholesterol (mmol/l); LDL=Low-density Lipoprotein (mmol/l); HDL=High-density Lipoprotein (mmol/l); Triglycerides in (mmol/l). For effect strengths Cohen’s ω for sex. SES category (%) und BMI category (%) was used, for lipid parameter eta².

Table 2: Characterization of participants in the subgroups ADHD with and without Methylphenidate intake at KiGGS follow-up after matching.

|  | **ADHD group (n = 258)**  **Follow-Up after matching** | | | |
| --- | --- | --- | --- | --- |
|  | Participants with MPH (n=26) | Participants without MPH (n = 232) | p-value | Effect size |
| Age | 21.85±2.68 | 21.54±2.96 |  |  |
| Sex (%), female | 11.54 | 30.17 | 0.068 | 0.097 |
| BMI | 25.30±5.57 | 24.89±5.47 |  |  |
| BMI category (%) |  |  | 0.103 | 0.136 |
| Underweight (BMI < 18.5) | 3.85 | 4.31 |  |  |
| Normal (18.5 ≤ BMI < 25.0) | 57.69 | 56.47 |  |  |
| Overweight (25.0 ≤ BMI < 30.0) | 23.08 | 21.98 |  |  |
| Obese (BMI > 30.0) | 15.38 | 17.24 |  |  |
| Heart rate (bpm) | 69.81±11.39 | 75.67±13.12 |  |  |
| Total cholesterol (mmol/l) | 4.37±0.76 | 4.60±0.96 | 0.00 |  |
| LDL (mmol/l) | 2.50±0.70 | 2.62±0.75 | 0.00 |  |
| HDL (mmol/l) | 1.27±0.25 | 1.36±0.31 | 0.00 |  |
| Triglycerides (mmol/l) | 1.45±1.22 | 1.43±0.96 | 0.00 |  |

Annotations: age (years); sex (percentage of female participants); SDQ-H/I=Strengths and Difficulties Questionnaire Subscale Hyperactivity/Inattention; SES=socioeconomic status; BMI=Body Mass Index (kg/m²). Heart rate (bpm = beats per minutes); Total Cholesterol (mmol/l); LDL=Low-density Lipoprotein (mmol/l); HDL=High-density Lipoprotein (mmol/l); Triglycerides in (mmol/l). For effect strengths Cohen’s ω for sex. SES category (%) und BMI category (%) was used, for lipid parameter eta². Note: there is no sufficient data available about SES and SDQ in this subgroup

Figure 1. Group participants and matching flowchart.


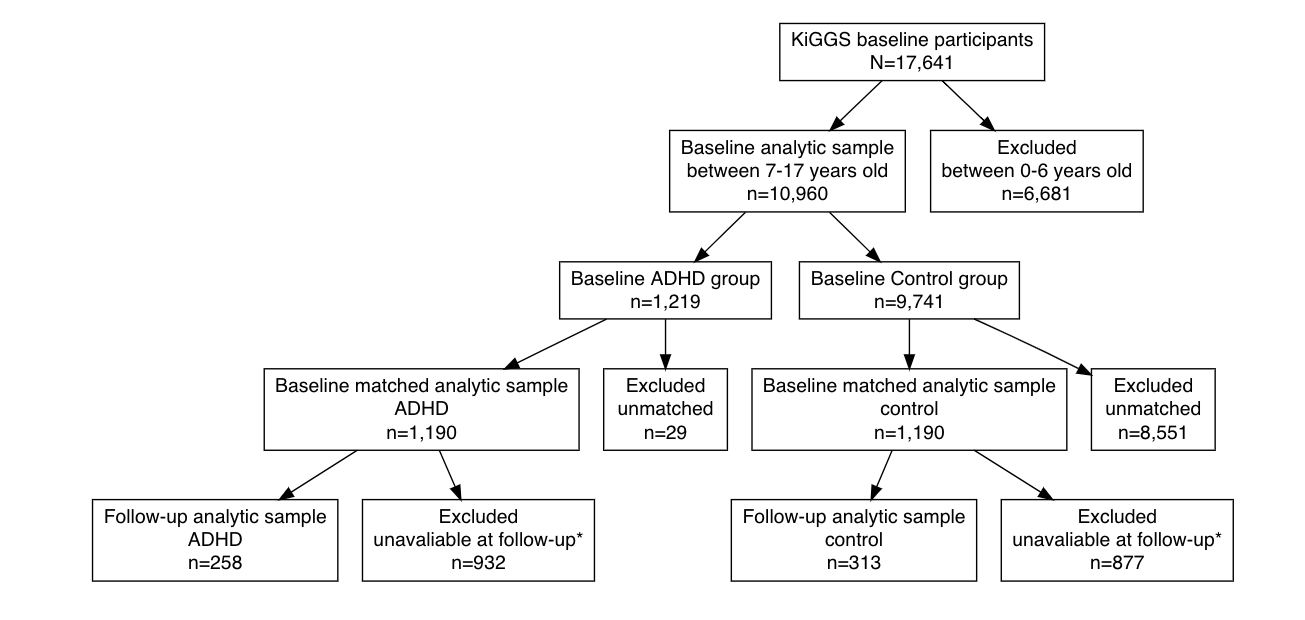


Note: * Participants were excluded due to lack of lipid measurements at the ten-year follow up.

Table 3. Additional analysis. Comparison of BMI and lipids in ADHD and controlgroup at follow-up with the subgroup normal and underweight.

|  | BMI group 1  (n=361) | BMI group 2  (n=210) | p-value | effect size |
| --- | --- | --- | --- | --- |
| Total cholesterol (mmol/l) | 4.43 ± 0.90 | 4.73 ± 0.93 | < 0.001 | 0.322 |
| LDL (mmol/l) | 2.45 ± 0.70 | 2.79 ± 0.74 | < 0.001 | 0.477 |
| HDL (mmol/l) | 1.41 ± 0.31 | 1.26 ± 0.28 | < 0.001 | 0.497 |
| Triglycerides (mmol/l) | 1.24 ± 0.80 | 1.60 ± 1.06 | < 0.001 | 0.398 |

Annotations: BMI=Body Mass Index (kg/m²), group 1: BMI < 25.0, category underweight or normal, group 2: BMI $\geq$ 25.0, category overweight or obese; Total cholesterol (mmol/l); LDL=low-density lipoprotein (mmol/l); HDL=high-density lipoprotein (mmol/l); triglycerides in (mmol/l). For effect sizes, Cohen’s ω for sex, SES category (%) und BMI category (%) was used, for all others Cohen’s d.

Table 4. Additional analysis. Comparison of BMI and lipids in ADHD and controlgroup at follow-up with the subgroup overweight and obese

|  | BMI group 1 | | | |  | BMI group 2 | | | |
| --- | --- | --- | --- | --- | --- | --- | --- | --- | --- |
|  | ADHD  (n=157) | Control  (n=204) | p-value | effect size |  | ADHD  (n=101) | Control  (n=109) | p-value | effect size |
| Total cholesterol (mmol/l) | 4.45 ± 0.94 | 4.42 ± 0.88 | 0.792 | 0.028 |  | 4.77 ± 0.93 | 4.69 ± 0.93 | 0.565 | 0.080 |
| LDL (mmol/l) | 2.47 ± 0.72 | 2.44 ± 0.68 | 0.723 | 0.038 |  | 2.84 ± 0.73 | 2.75 ± 0.75 | 0.400 | 0.116 |
| HDL (mmol/l) | 1.41 ± 0.30 | 1.41 ± 0.32 | 0.979 | 0.003 |  | 1.25 ± 0.28 | 1.27 ± 0.28 | 0.538 | 0.085 |
| Triglycerides (mmol/l) | 1.30 ± 0.91 | 1.20 ± 0.71 | 0.239 | 0.125 |  | 1.64 ± 1.06 | 1.27 ± 0.28 | 0.611 | 0.070 |

Annotations: BMI=Body Mass Index (kg/m²), group 1: BMI < 25.0, category underweight or normal, group 2: BMI $\geq$ 25.0, category overweight or obese; Total cholesterol (mmol/l); LDL=low-density lipoprotein (mmol/l); HDL=high-density lipoprotein (mmol/l); triglycerides in (mmol/l). For effect sizes, Cohen’s ω for sex, SES category (%) und BMI category (%) was used, for all others Cohen’s d.

References

Banaschewski T, Hohmann S, Millenet Sea (2017) Langfassung der interdisziplinären evidenz- und konsensbasierten (S3) Leitlinie “Aufmerksamkeitsdefizit-/ Hyperaktivitätsstörung (ADHS) im Kindes-, Jugend- und Erwachsenenalter”. AWMF-Registrierungsnummer 028-045

Charach G, Kaysar N, Grosskopf I, Rabinovich A, Weintraub M (2009) Methylphenidate has positive hypocholesterolemic and hypotriglyceridemic effects: new data. J Clin Pharmacol 49:848–851. <https://doi.org/10.1177/0091270009336736>

Deeb A, Attia S, Mahmoud S, Elhaj G, Elfatih A (2018) Dyslipidemia and Fatty Liver Disease in Overweight and Obese Children. J Obes. <https://doi.org/10.1155/2018/8626818>

Kase BE, Rommelse N, Chen Q, Li L, Andersson A, Du Rietz E, Vos M, Cortese S, Larsson H, Hartman CA (2021) Longitudinal Associations Between Symptoms of ADHD and BMI From Late Childhood to Early Adulthood. Pediatrics 147(6). <https://doi.org/10.1542/peds.2020-036657>

Knopf H (2007) Arzneimittelanwendung bei Kindern und Jugendlichen. Erfassung und erste Ergebnisse beim Kinder- und Jugendgesundheitssurvey (KiGGS). Bundesgesundheitsbl - Gesundheitsforsch - Gesundheitsschutz 50(5-6):863–870. <https://doi.org/10.1007/s00103-007-0249-z>

Korsten-Reck U, Kromeyer-Hauschild K, Korsten K, Baumstark MW, Dickhuth H-H, Berg A (2008) Frequency of secondary dyslipidemia in obese children. Vasc Health Risk Manag 4(5):1089–1094. <https://doi.org/10.2147/vhrm.s2928>

Robert Koch-Institut (RKI), Bundeszentrale für gesundheitliche Aufklärung Erkennen – Bewerten – Handeln: Zur Gesundheit von. Arzneimittelkonsum:145–175. <https://www.rki.de/DE/Content/Gesundheitsmonitoring/Studien/Kiggs/Basiserhebung/GPA_Daten/Arzneimittel.pdf?__blob=publicationFile>. Accessed 07 Sep 2022
